# High On/Off Ratio Spintronic Multi‐Level Memory Unit for Deep Neural Network

**DOI:** 10.1002/advs.202103357

**Published:** 2022-02-20

**Authors:** Kun Zhang, Xiaotao Jia, Kaihua Cao, Jinkai Wang, Yue Zhang, Kelian Lin, Lei Chen, Xueqiang Feng, Zhenyi Zheng, Zhizhong Zhang, Youguang Zhang, Weisheng Zhao

**Affiliations:** ^1^ Fert Beijing Research Institute MIIT Key Laboratory of Spintronics School of Integrated Circuit Science and Engineering Beihang University Beijing 100191 P. R. China; ^2^ Beihang‐Goertek Joint Microelectronics Institute Qingdao Research Institute Beihang University Qingdao 266101 P. R. China; ^3^ Beihang Hangzhou Innovation Institute Yuhang Xixi Octagon City, Yuhang District Hangzhou 310023 P. R. China; ^4^ Nanoelectronics Science and Technology Center Hefei Innovation Research Institute Beihang University Hefei 230013 P. R. China

**Keywords:** deep neural network, diode, high on/off ratio, magnetic tunnel junction, multi‐level memory unit

## Abstract

Spintronic devices are considered as one of the most promising technologies for non‐volatile memory and computing. However, two crucial drawbacks, that is, lack of intrinsic multi‐level operation and low on/off ratio, greatly hinder their further application for advanced computing concepts, such as deep neural network (DNN) accelerator. In this paper, a spintronic multi‐level memory unit with high on/off ratio is proposed by integrating several series‐connected magnetic tunnel junctions (MTJs) with perpendicular magnetic anisotropy (PMA) and a Schottky diode in parallel. Due to the rectification effect on the PMA MTJ, an on/off ratio over 100, two orders of magnitude higher than intrinsic values, is obtained under proper proportion of alternating current and direct current. Multiple resistance states are stably achieved and can be reconfigured by spin transfer torque effect. A computing‐in‐memory architecture based DNN accelerator for image classification with the experimental parameters of this proposal to evidence its application potential is also evaluated. This work can satisfy the rigorous requirements of DNN for memory unit and promote the development of high‐accuracy and robust artificial intelligence applications.

## Graphical abstract

A spintronic multi‐level memory unit (MLMU) is developed by integrating series‐connected magnetic tunnel junctions and a Schottky diode in parallel. Multiple stable resistance states configured by spin transfer torque effect combining with high on/off ratio are achieved. Based on the parameters of MLMU, a computing‐in‐memory based deep neural network accelerator for image classification is evaluated to evidence its application potential.

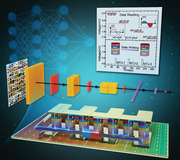

## 1 Introduction

Nowadays, neuromorphic computing systems, such as deep neural network (DNN), have attracted large amounts of attention and been widely used in artificial intelligence (AI) areas, such as image classification, speech recognition, and automatic driving.^[^ ^1^, ^2^, ^3^, ^4^, ^5^ ^]^ The computing‐in‐memory (CiM) architecture based on emerging non‐volatile random access memory (RAM), such as phase‐change RAM,^[^ ^6^, ^7^, ^8^ ^]^ ferroelectric RAM,^[^ ^9^, ^10^, ^11^ ^]^ resistive RAM,^[^ ^12^, ^13^, ^14^ ^]^ and spin‐transfer torque magnetic RAM (STT‐MRAM),^[^ ^15^, ^16^, ^17^, ^18^ ^]^ injects new vitality to the development of DNN, which can effectively improve the computation capability and reduce energy consumption.^[^ ^19^, ^20^, ^21^, ^22^, ^23^, ^24^ ^]^ Among them, STT‐MRAM has been widely applied in aerospace field and consumer electronic product in the form of embedded memory thanks to its high speed, low power consumption, scalability, and infinite endurance.^[^ ^25^, ^26^, ^27^ ^]^ However, the binary property and relatively low tunnel magnetoresistance (TMR) ratio (i.e., ≈2.5)^[^ ^28^ ^]^ lead to worse learning accuracy and more redundancy for DNN applications.^[^ ^29^ ^]^ Several solutions, such as perpendicular‐stacked and series‐connected multiple MTJs,^[^ ^30^, ^31^, ^32^, ^33^, ^34^ ^]^ current‐induced domain wall motion,^[^ ^35^, ^36^, ^37^ ^]^ and magnetic skyrmions manipulation^[^ ^38^ ^]^ are creatively utilized to overcome the binary limitation of STT‐MRAM. However, the issue of low on/off ratio has remained unsolved. Meanwhile, their poor compatibility and reliability as well as high fabrication cost make them far away from practical applications in the next few years. In this context, novel spintronic schemes possessing stable multiple resistance states, high on/off ratio, and excellent compatibility with STT‐MRAM are greatly required for improving the performance of CiM‐based DNN.

**Figure 1:**
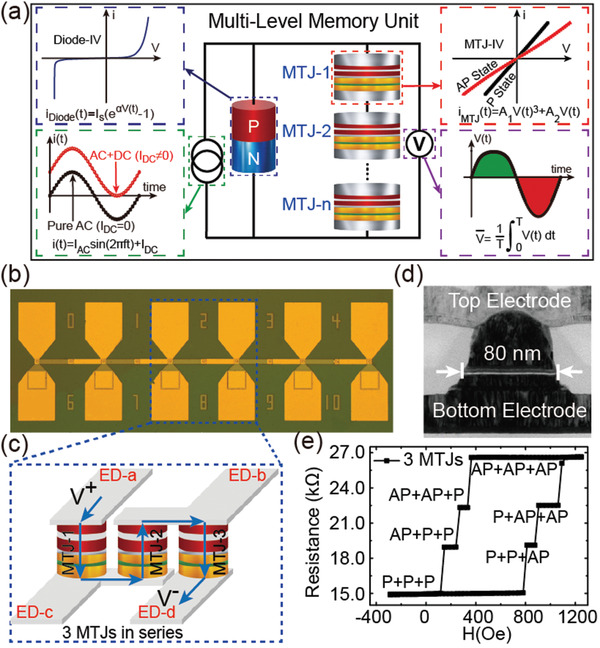
a) Scheme of the proposed spintronic MLMU, where the expressions of constructed components and measurement methods are also presented. b) Optical image of the fabricated PMA MTJ chain, in which varying numbers of MTJs can be connected in series. c) Schematic of three series‐connected PMA MTJs. d) TEM image of an 80‐nm‐diameter PMA MTJ pillar. e) *R–H* curve of three series‐connected PMA MTJs under DC = 0.5 µA.

In this work, we propose a spintronic multi‐level memory unit (MLMU) by integrating a chain of perpendicular magnetic anisotropy magnetic tunnel junctions (PMA MTJs) and a Schottky diode in parallel, as schematically shown in **Figure** **1**a. First, the series PMA MTJs with designed interconnected electrodes can not only provide stable multiple resistance states, but also be configured through spin transfer torque (STT) effect. Moreover, due to the rectification effect of Schottky diode on the PMA MTJs, the applied alternating current (AC) could be rectified to MTJ‐state‐dependent direct current (DC) voltage. Over 100 on/off ratio, two orders of magnitude higher than intrinsic values, can be realized under proper AC/DC proportion. The working mode of the MLMU, including data writing based on STT effect and data reading with high on/off ratio, is experimentally demonstrated. We further leverage the experimental results to perform architecture‐level simulations for a CiM based DNN accelerator toward image classification, in which high accuracy is presented. This state‐of‐the‐art demonstration can promote the development of spintronic devices and inspire more high‐performance AI applications.

## 2 Results and Discussions

### 2.1 PMA MTJs Chain and Multiple Resistance States

In order to realize multiple resistance states, we fabricate a chain composed of a number of 80‐nm‐diameter PMA MTJs by using standard up‐to‐down process, mainly including electron beam lithography and argon ion beam etching. As presented in Figure 1b, interconnection electrodes are specially designed to implement series connections of any number of MTJs. Figure 1c shows the detailed schematic of three neighboring MTJs in the chain. By charging electrode‐a (ED‐a) and electrode‐d (ED‐d), three MTJs are connected in series. The transmission electron microscopy (TEM) image of a PMA MTJ pillar demonstrates the high quality of the device fabrication as shown in Figure 1d.

Figure 1e plots the junction resistance varying with magnetic field (*R–H* curve) of three series‐connected MTJs. Four stable resistance states including “P + P + P,” “AP + P + P”/”P + P + AP,” “AP + AP + P”/”P + AP + AP,” and “AP + AP + AP” are observed (“P” and “AP” respectively represent parallel and antiparallel states of a single MTJ). It is noteworthy that we artificially adopt three MTJs with different coercive fields to clearly demonstrate the multi‐level operation. For realistic application, the MTJs can be highly uniform and we can utilize STT effect to configure the resistance states of each MTJ through charging corresponding electrodes. For example, by charging electrode‐c (ED‐c) and electrode‐b (ED‐b), we could locally set the state of MTJ‐2. From the measurement results, the maximum on/off ratio considering the difference between “AP + AP + AP” and “P + P + P” states is about 0.8, which can only be comparable to the TMR ratio of a single MTJ in the chain. This confirms that realizing multi‐level operation through pure connection of MTJs is not sufficient for further DNN application.

### 2.2 On/Off Ratio Amplification

**Figure 2:**
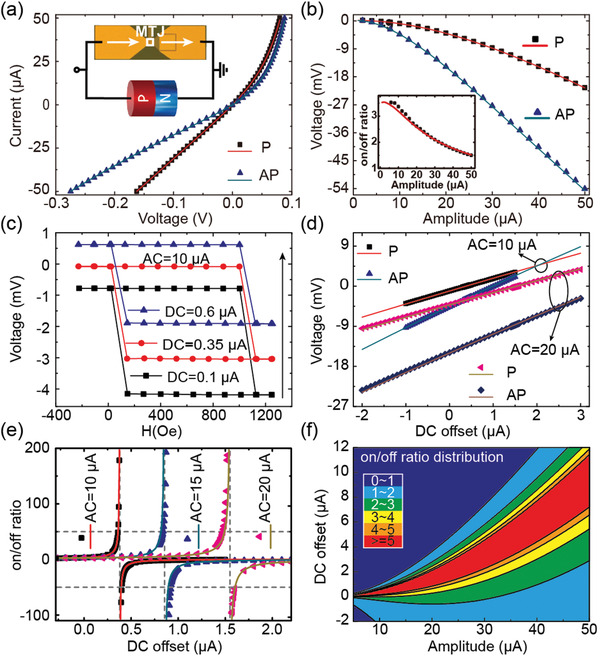
a) Measured and calculated *I–V* curve of the combined device. Inset shows the schematic of combined device. b) Measured and calculated rectification voltage versus AC amplitude. Inset shows the deduced on/off ratio. c) Dependence of rectification voltage on magnetic field under AC = 10 µA and various DC offsets. d) Measured and calculated rectification voltage as a function of DC offset with different AC amplitudes. e) Measured and calculated on/off ratio as a function of DC offset and AC amplitude. In (a), (b), (d), and (e), the scattering points are experimental data and the lines are theoretical simulation results. f) Distribution of the on/off ratio under different AC and DC components. The colors represent the absolute values of the on/off ratio.

To amplify the on/off ratio and well describe the amplification mechanism, a PMA MTJ and a Schottky diode are integrated in parallel as schematically illustrated in the inset of **Figure** **2**a. The basic transport properties of the discrete components (i.e., MTJ and Schottky diode) can be found in Section S1, Supporting Information. Figure 2a demonstrates the current–voltage (*I–V*) curves of the combined device for P and AP states. Significant asymmetry of positive and negative branches of the *I–V* curves indicates the existence of rectification behavior. Only AC is then applied to the proposed device and measure the generated rectification voltage. In this process, the current flowing through the diode would be rectified to DC voltage. Hence, with the increasing AC amplitude, the shunting current through the diode increases, resulting in the nonlinear augment of the rectification voltage as shown in Figure 2b. Moreover, the resistance states of MTJs can modulate the shunting current through the diode. Under a fixed AC amplitude, the shunting current flowing through diode increases for the transition from P state to AP state, leading to an enlargement in the absolute value of rectification voltage. The deduced on/off ratio decrease slowly as shown in the inset of Figure 2b. The on/off ratio here is always defined as $\frac{\bar{V_{\mathrm{AP}}\;}-\;\bar{V_{P}}}{\bar{V_{P}}}$, where $\bar{V_{\mathrm{AP}}}$ ($\bar{V_{P}}$) represents the rectification voltage under AP (P) state. Maximum on/off ratio of 3.5 is observed, which is much larger than the TMR value of the single MTJ (i.e., 0.8).

These results indicate that the transport properties of the combined device can be manipulated by simultaneous application of AC and DC. In this case, the AC‐induced rectified voltage and DC‐induced voltage drop are measured together. With the increasing DC offset, the detected voltages shift vertically while their forms remain nearly unchanged as shown in Figure 2c. As a result, the measured rectification voltage under P state ($\bar{V_{P}}$) can be tuned to nearly zero under fixed AC amplitude by controlling the DC offset, which contributes to the great enhancement of on/off ratio. Figure 2d plots the DC offset dependent rectification voltage under different AC components. With the augmentation of AC amplitude, the DC offset to set $\bar{V_{P}}=\;0$ also increases. As summarized in Figure 2e, over 100 on/off ratio can be obtained through varying DC offset. The DC offset range exhibiting high on/off ratio enlarges with the increasing AC amplitude.

Through combining the mentioned equations in Figure 1a, including the transport properties of the constructed components (i.e., diode and MTJ) and the expressions of the applied AC with DC offset and measured rectification voltage, we can figure out the magneto‐transport properties of the combined devices under different states, which is in good agreement with the experimental observations as presented in Figure 2a,b,d,e. See Section S2, Supporting Information for the detailed establishment process of the physical model. According to the physical model, we can figure out the intermediate results to further clarify the enhancement mechanism of the on/off ratio. Furthermore, we can predict the on/off ratio distribution. Figure 2f shows the AC amplitude and DC offset dependence of the on/off ratio. In a wide amplitude range, the dependence presents a fan‐shaped distribution. As a result, high on/off ratios can be achieved in a wide range of configurations. And with the increase of AC amplitude and DC offset, the area representing large on/off ratios (>5, red area) expands.

### 2.3 Spintronic MLMU and Its Working Mode

**Figure 3:**
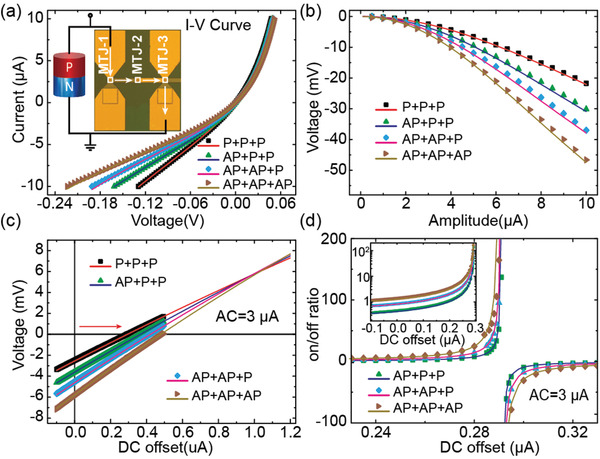
a) Measured and calculated *I–V* curves of the MLMU. The inset shows schematic of the four‐state MLMU. b) Measured and calculated rectification voltage of each state versus AC amplitude. c) Measured and calculated rectification voltage of each state modulated by DC offsets under AC = 3 µA. d) On/off ratio of the MLMU with AC and DC modulation. Inset: positive branch under logarithmic ordinate. In these figures, the scattering points are experimental data and the lines are theoretical simulation results.

To combine the advantages of multiple resistance and high on/off ratio, we design and construct a spintronic MLMU by connecting a Schottky diode and a PMA MTJ chain in parallel, as shown in the inset of **Figure** **3**a. The rectification effect is then superimposed on the multiple resistance states phenomenon. We first characterize its DC transport properties. Figure 3a shows the *I–V* curves of our spintronic MLMU under different resistance states, which exhibit obvious asymmetry between positive and negative branches. For the positive branches, the *I–V* curves under different states nearly overlap with each other because of the significant shunting effect of diode under positive voltage. For the negative branches, the *I–V* curves separate clearly due to the off state of diode under negative voltage. We then apply a pure AC to the spintronic MLMU. Figure 3b presents the rectification voltage varying with the AC amplitude. The rectification voltage increases nonlinearly with the augment of AC amplitude under different resistance states.

We investigate the magneto‐transport property of the spintronic MLMU under the case that both AC and DC components are simultaneously applied. Figure 3c presents the DC offset dependence of measured voltage under AC = 3 µA. The deviations from the coordinate origin are because that the AC‐induced rectification voltage is superimposed on the DC‐induced voltage drop. With this modulation, high on/off ratios (e.g., >100) can be achieved under AC = 3 µA and DC = 0.29 *μ*A as demonstrated in Figure 3d. The inset of Figure 3d shows the positive branches under logarithmic ordinate, from which we can clearly obtain the variation trend of on/off ratio under different states away from 0.29 *μ*A. Based on theoretical model, we can also well describe the characterizations of the MLMU.

**Figure 4:**
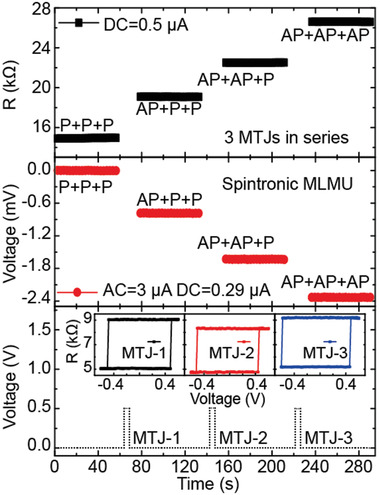
Operating process of the 4 MLMU including data writing based on STT effect and data reading with high on/off ratio. Inset: STT‐induced switching of each MTJ. Bottom figure frame: the independently applied pulse voltage on different MTJs. Middle figure frame: the measured rectification voltage of spintronic MLMU under different states with AC = 3 µA and DC = 0.29 µA. Top figure frame: the corresponding resistance states of the three series‐connected MTJs under DC = 0.5 µA.

To demonstrate the working mode of our proposed MLMU in realistic applications, we carry out a complete data operating process including data writing based on STT effect and data reading with high on/off ratio. As demonstrated in **Figure** **4**, three PMA MTJs integrated in the MLMU can be respectively switched from P state to AP state by locally applying a voltage pulse of 0.5 V to the corresponding electrodes. The inset shows the STT‐induced switching of each MTJ. The top figure frame illustrates the resistance states of the three series MTJs measured under DC = 0.5 *μ*A, which is consistent with the measured *R–H* curve in Figure 1e. As a result, through the interconnected electrodes and the current‐induced STT effect, the resistance states of each MTJ can be flexibly rewritten, and the resistance states of the MLMU are linearly updated without resetting process. Corresponding to different resistance states, multi‐level rectification voltages are stably detected under AC = 3 µA and DC = 0.29 *μ*A. We also construct a three‐state MLMU by integrating two‐series MTJs and a diode in parallel. The detailed transport properties and complete operation process are presented in Section S3, Supporting Information.

From the perspective of practical application, the integration scheme and integration density of the proposed spintronic MULU should be discussed. At first, the proposed spintronic MLMU can be integrated in a crossbar array structure. Through the selection of bit line, word line, and corresponding transistors, the STT‐based data writing and data reading with high on/off ratio can be implemented. Here, the input signals can be encoded into different proportions of AC and DC components while the output multivalued voltage can be sensed out by utilizing an analog‐to‐digital conversion circuit or other feasible schemes. The detailed design scheme of the crossbar array as well as the encode and read scheme can be found in Section S4, Supporting Information. In this design scheme, a basic structure of one transistor and one MTJ is utilized. Therefore, the integration density of our scheme is comparable with that of MRAM. In addition, through connecting more MTJs in series and configuring the junction resistance of each MTJ, the resistance states provided by one MTJ (i.e., the integration density of our MLMU) can be extremely improved (see Section S5, Supporting Information).

### 2.4 CiM based DNN and Performance Evaluation

**Figure 5:**
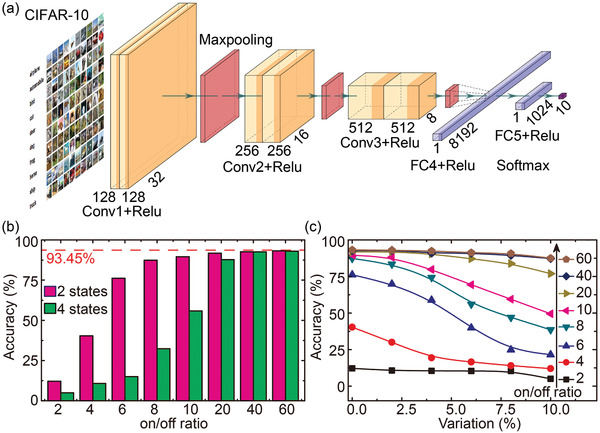
a) Schematic of the VGG‐8 model used for classification of images from CIFAR‐10 dataset. b) Classification accuracy versus on/off ratio for two and four states per unit. c) Impact of device/state variations on classification accuracy for different on/off ratios.

In order to reveal the system‐level performance of the spintronic MLMU for DNN application, we treat it as a non‐volatile memory unit that is the main component of CiM architecture based DNN accelerator. Then a simulation platform MNSim^[^ ^39^ ^]^ is utilized to evaluate its performance. In our evaluation, VGG‐8 is used as a case study to classify the 32 × 32‐colored images from CIFAR‐10 dataset^[^ ^40^ ^]^ as shown in **Figure** **5**a. VGG‐8 is trained offline by Pytorch using GPU that could achieve an inference accuracy of 93.45%. In the simulator, the neural network parameters (e.g., weight, bias) are expressed with the format of INT‐8 that are stored in several *n*‐state memory units. For example, if one MLMU has two states, eight units are needed to store one parameter. If one MLMU has four states, then four units are needed. In our case, for large *n*, multiple MTJs can share one diode, which is beneficial for high‐density integration. For other neuromorphic computing architectures, we can only use one memory unit to store one parameter. Therefore, the state number of one memory unit determines the precision of the parameters, and then significantly influences the inference accuracy.^[^ ^41^ ^]^

The simulation results show that the inference accuracy highly depends on the on/off ratio and state/device variation. As shown in Figure 5b with the increasing on/off ratio, the inference accuracy increases. For two‐state MLMU, the accuracy reaches the software baseline (93.45%) when the on/off ratio is larger than 10. For four‐state MLMU, the on/off ratio should be larger than 20. Meanwhile, the reliability of the implemented high on/off ratio is also critical for practical application. As shown in Figure 2f, the area exhibiting high on/off ratio enlarges with the increasing AC and DC component, which means that the proposed MLMU possesses high robustness against to fluctuations induced by parasitic IR drop or other non‐idealities. The results shown in Figure 5c indicate that high on/off ratio is much more important to inference accuracy when device variation is considered. In the case of relatively low on/off ratios, high variations would lead to a remarkable degradation of inference accuracy, which further highlights the importance of the on/off ratio. In brief, high on/off ratio can improve not only the inference accuracy, but also the robustness against to state/device variation. Something should be noted that the dependence of inference accuracy on on/off ratio become stronger with the increasing state number. This is because that the increasing states number would reduce the difference between neighboring states for a constant on/off ratio. These results and discussions indicate that the proposed MLMU featuring high on/off ratio and reliable multi‐level operation is suitable for CiM architecture based DNN accelerator. In addition, thanks to the non‐volatility, the energy consumption of DNN accelerator based on our unit would be dramatically reduced compared with that based on static RAM.

## 3 Conclusion

In summary, we construct a spintronic MLMU providing high on/off ratio and reliable multiple resistance states by integrating a chain of PMA MTJs and Schottky diode. The complete data operating process oriented to realistic systems and the performance evaluation on a DNN accelerator based on CiM architecture have also been implemented to confirm the prospect of the constructed MLMU. This work would break the bottlenecks of conventional spintronic devices and inspire more high‐performance AI applications.

## 4 Experimental Section

### Device Preparation

The double MgO/CoFeB‐interface MTJs with enhanced PMA used in this study were fabricated through the standard top–down fabrication approach. From the substrate side, Ta(5)/Ru(30)/Ta(0.7)/Pt(1.5)/[Co(0.5)/Pt(0.2)]_6_/Co(0.6)/Ru(0.8)/Co(0.6)/ [Pt(0.2)/Co(0.5)]_3_/W(0.25)/CoFeB(1.0)/MgO(0.8)/CoFeB(1.3)/W(0.2)/CoFeB(0.5)/MgO(0.75)/Ta(3.0) multilayer stacks (numbers in parenthesis represent layer thicknesses in nm) were deposited on a thermally oxidized silicon substrate using a Singulus magnetron sputtering machine. After annealing at 390 °C for an hour in high vacuum (about 3.75 × 10^−10^ torr), the multilayer films were patterned into circular nano‐pillars using electron beam lithography and argon ion beam etching techniques. The bottom W/CoFeB/MgO layer was pinned by a synthetic antiferromagnetic layer of [Co/Pt]_6_/Ru/[Pt/Co]_3_ to form a reference layer. The MgO/CoFeB/W/CoFeB/MgO multilayer with double MgO/CoFeB interfaces was the free layer, which possessed a considerable thermal stability factor and comparable critical current density compared to the single‐interface free layer. The rectification component used in the experiments was a Schottky diode with a series number of BAT85.

### Electrical Measurement

All the electrical transport properties were measured at room temperature in the Lake Shore CRX‐VF cryogenic probe station with the applied magnetic field perpendicular to the plane of the silicon substrate. In order to remove the electrode resistance, four‐point method was used to characterize the transport properties of the MTJs. For the diode and MLMU, two‐point method was used. During the measurement, a Keithley 6221 source meter was used to supply the DC and AC with various DC offsets at fixed frequency of 1 kHz while a Keithley 2182A voltage meter was used to measure the DC voltage drop and rectification voltage.

### DNN Simulations

The CIFAR‐10 dataset consisted of 60 000 32 × 32 color images in 10 classes (e.g., airplane, ship, dog, etc.), with 6000 images per class. There were 50 000 training images and 10 000 test images. The dataset was divided into five training batches and one test batch, each with 10 000 images. For each calculation of classification accuracy, the test batch contains exactly1000 randomly‐selected images from each class.

### Statistical Analysis

For experimental results, each point was obtained from an average of ten measurements. For DC transport property measurement (i.e., Figure 1e, inset and top figure frame of Figure 4), the applied current (*I*) and measured voltage (*V*) were recorded to calculate the resistance (*R*) of the series‐connected MTJs by the equation, *R*  = *V*/*I*. Origin Software was used for data processing and analysis.

## Conflict of Interest

The authors declare no conflict of interest.

## Supporting information

Supporting Information

## Data Availability

The data that support the findings of this study are available from the corresponding author upon reasonable request.

## References

[1] A. Krizhevsky , I. Sutskever , G. E. Hinton , Commun. ACM 2017, 60, 84.

[2] G. Hinton , L. Deng , D. Yu , G. Dahl , A.‐r. Mohamed , N. Jaitly , A. Senior , V. Vanhoucke , P. Nguyen , T. Sainath , B. Kingsbury , IEEE Signal Process. Mag. 2012, 29, 82.

[3] C. Chen , A. Seff , A. Kornhauser , J. Xiao , 2015 IEEE Int. Conf. Computer Vision (ICCV), IEEE, Santiago, Chile 2015, p. 2722.

[4] A. B. Nassif , I. Shahin , I. Attili , M. Azzeh , K. Shaalan , IEEE Access 2019, 7, 19143.

[5] P. M. Kebria , A. Khosravi , S. M. Salaken , S. Nahavandi , IEEE/CAA J. Autom. Sin. 2020, 7, 82.

[6] Y. T. Liu , X. B. Li , H. Zheng , N. K. Chen , X. P. Wang , X. L. Zhang , H. B. Sun , S. Zhang , Adv. Funct. Mater. 2021, 31, 2009803.

[7] V. Joshi , M. L. Gallo , S. Haefeli , I. Boybat , S. R. Nandakumar , C. Piveteau , M. Dazzi , B. Rajendran , A. Sebastian , E. Eleftheriou , Nat. Commun. 2020, 11, 2473. 32424184 10.1038/s41467-020-16108-9 PMC7235046

[8] D. Kuzum , R. G. Jeyasingh , B. Lee , H. S. Wong , Nano Lett. 2012, 12, 2179. 21668029 10.1021/nl201040y

[9] S. Majumdar , H. Tan , Q. H. Qin , S. van Dijken , Adv. Electron. Mater. 2019, 5, 1800795.

[10] B. Li , S. Li , H. Wang , L. Chen , L. Liu , X. Feng , Y. Li , J. Chen , X. Gong , K. W. Ang , Adv. Electron. Mater. 2020, 6, 2000760.

[11] M. K. Kim , J. S. Lee , Nano Lett. 2019, 19, 2044. 30698976 10.1021/acs.nanolett.9b00180

[12] R. Waser , M. Aono , Nat. Mater. 2007, 6, 833. 17972938 10.1038/nmat2023

[13] Q. Wang , X. Wang , S. H. Lee , F.‐H. Meng , W. D. Lu , 2019 IEEE Int. Electron Devices Meeting, IEEE, San Francisco, CA 2019, p. 14.4.1.

[14] P. Yao , H. Wu , B. Gao , J. Tang , Q. Zhang , W. Zhang , J. J. Yang , H. Qian , Nature 2020, 577, 641. 31996818 10.1038/s41586-020-1942-4

[15] J. Grollier , D. Querlioz , K. Y. Camsari , K. Everschor‐Sitte , S. Fukami , M. D. Stiles , Nat. Electron. 2020, 3, 360. 10.1038/s41928-019-0360-9 PMC7754689 33367204

[16] J. Torrejon , M. Riou , F. A. Araujo , S. Tsunegi , G. Khalsa , D. Querlioz , P. Bortolotti , V. Cros , K. Yakushiji , A. Fukushima , H. Kubota , S. Yuasa , M. D. Stiles , J. Grollier , Nature 2017, 547, 428. 28748930 10.1038/nature23011 PMC5575904

[17] A. D. Patil , H. Hua , S. Gonugondla , M. Kang , N. R. Shanbhag , IEEE Int. Symp. Circuits and System (ISCAS), IEEE, Piscataway 2019.

[18] M.‐H. Wu , M.‐C. Hong , C.‐C. Chang , P. Sahu , J.‐H. Wei , H.‐Y. Lee , S.‐S. Sheu , T.‐H. Hou 2019 Symp. VLSI Technology, IEEE, Kyoto, Japan 2019, p. T34.

[19] S. Yu , Proc. IEEE 2018, 106, 260.

[20] S. Jain , A. Ranjan , K. Roy , A. Raghunathan , IEEE Trans. Very Large Scale Integr. VLSI Syst. 2018, 26, 470.

[21] T. Kim , H. Kim , J. Kim , J.‐J. Kim , IEEE Electron Device Lett. 2017, 38, 1228. 10.1109/LED.2017.2696002 PMC5590659 28890601

[22] B. Chen , F. Cai , J. Zhou , W. Ma , P. Sheridan , W. D. Lu , 2015 IEEE. Int. Electron Devices Meeting (IEDM) , IEEE, Piscataway 2015, p. 17.5.1.

[23] D. B. Strukov , G. S. Snider , D. R. Stewart , R. S. Williams , Nature 2008, 453, 80. 18451858 10.1038/nature06932

[24] H. S. Wong , S. Salahuddin , Nat. Nanotechnol. 2015, 10, 191. 25740127 10.1038/nnano.2015.29

[25] A. D. Kent , D. C. Worledge , Nat. Nanotechnol. 2015, 10, 187. 25740126 10.1038/nnano.2015.24

[26] S. H. Kang , C. Park , 2017 IEEE. Int. Electron Devices Meeting (IEDM), IEEE, San Francisco, CA 2017, p. 38.2.1.

[27] G. Wang , Y. Zhang , J. Wang , Z. Zhang , K. Zhang , Z. Zheng , J.‐O. Klein , D. Ravelosona , Y. Zhang , W. Zhao , IEEE Trans. Electron Devices 2019, 66, 2431.

[28] M. Wang , W. Cai , K. Cao , J. Zhou , J. Wrona , S. Peng , H. Yang , J. Wei , W. Kang , Y. Zhang , J. Langer , B. Ocker , A. Fert , W. Zhao , Nat. Commun. 2018, 9, 671. 29445186 10.1038/s41467-018-03140-z PMC5813193

[29] A. Pal Chowdhury , P. Kulkarni , M. Nazm Bojnordi , J. Low Power Electron. Appl. 2018, 8, 38.

[30] D. Zhang , L. Zeng , K. Cao , M. Wang , S. Peng , Y. Zhang , Y. Zhang , J. O. Klein , Y. Wang , W. Zhao , IEEE Trans. Biomed. Circuits Syst. 2016, 10, 828. 27214913 10.1109/TBCAS.2016.2533798

[31] J. Hong , X. Li , N. Xu , H. Chen , S. Cabrini , S. Khizroev , J. Bokor , L. You , Adv. Inell. Syst. 2020, 2, 2000143.

[32] T. Ishigaki , T. Kawahara , R. Takemura , K. Ono , K. Ito , H. Matsuoka , H. Ohno , 2010 Symp. VLSI Technology, IEEE, Honolulu, HI 2010, p. 47.

[33] Y. Zhang , L. Zhang , W. Wen , G. Sun , Y. Chen , 2012 IEEE/ACM Int. Conf. Computer‐Aided Design (ICCAD), IEEE, San Jose, CA 2012, p. 526.

[34] X. Fong , Y. Kim , R. Venkatesan , S. H. Choday , A. Raghunathan , K. Roy , Proc. IEEE 2016, 104, 1449.

[35] S. Lequeux , J. Sampaio , V. Cros , K. Yakushiji , A. Fukushima , R. Matsumoto , H. Kubota , S. Yuasa , J. Grollier , Sci. Rep. 2016, 6, 31510. 27539144 10.1038/srep31510 PMC4990964

[36] X. Zhang , W. Cai , M. Wang , B. Pan , K. Cao , M. Guo , T. Zhang , H. Cheng , S. Li , D. Zhu , L. Wang , F. Shi , J. Du , W. Zhao , Adv. Sci. 2021, 8, 2004645. 10.1002/advs.202004645 PMC8132064 34026457

[37] X. Lou , Z. Gao , D. V. Dimitrov , X. Tang , Appl. Phys. Lett. 2008, 93, 242502.

[38] K. M. Song , J.‐S. Jeong , B. Pan , X. Zhang , J. Xia , S. Cha , T.‐E. Park , K. Kim , S. Finizio , J. Raabe , J. Chang , Y. Zhou , W. Zhao , W. Kang , H. Ju , S. Woo , Nat. Electron. 2020, 3, 148.

[39] L. Xia , B. Li , T. Tang , P. Gu , P.‐Y. Chen , S. Yu , Y. Cao , Y. Wang , Y. Xie , H. Yang , IEEE Trans. CAD 2017, 37, 1009.

[40] A. Krizhevsk , Master Thesis, University of Toronto 2009.

[41] A. Mehonic , D. Joksas , W. H. Ng , M. Buckwell , A. J. Kenyon , Front. Neurosci. 2019, 13, 593. 31249502 10.3389/fnins.2019.00593 PMC6582938

