# Supplementary material for: High On/Off Ratio Spintronic Multi‐Level Memory Unit for Deep Neural Network
Source: Adv Sci (Weinh). 2022 Feb 20;9(13):2103357. doi: 10.1002/advs.202103357 (PMC9069383; doi:10.1002/advs.202103357)
Supplement: Supplementary file 1 — Supporting Information [file ADVS-9-2103357-s001.pdf]

ADVANCED SCIENCE
Open Access

Supporting Information

for *Adv. Sci.*, DOI 10.1002/advs.202103357

High On/Off Ratio Spintronic Multi-Level Memory Unit for Deep Neural Network

Kun Zhang, Xiaotao Jia, Kaihua Cao, Jinkai Wang, Yue Zhang, Kelian Lin, Lei Chen, Xueqiang Feng, Zhenyi Zheng, Zhizhong Zhang, Youguang Zhang and Weisheng Zhao*

Supporting Information

High On/Off Ratio Spintronic Multi-Level Memory Unit for Deep Neural Network

Kun Zhang, Xiaotao Jia, Kaihua Cao, Jinkai Wang, Yue Zhang, Kelian Lin, Lei Chen, Xueqiang Feng, Zhenyi Zheng, Zhizhong Zhang, Youguang Zhang, and Weisheng Zhao*

S1. Basic properties of discrete components

We measure the basic properties of discrete components, i.e., magnetic tunnel junction (MTJ) and Schottky diode, which can help to understand the working mechanism of our multi-level memory unit (MLMU). Fig. S1 (a) presents the out-of-plane (OOP) and in-plane (IP) magnetic hysteresis (M-H) loops of the multilayer stack for fabricating MTJ, exhibiting the excellent perpendicular magnetic anisotropy (PMA). The multiple flip steps of the OOP M-H curve reveal the relative magnetization orientation of the free layer, reference layer and synthesis antiferromagnetic layer.

As shown in Fig. S1 (b), the magnetic field dependent resistance (R-H) of the fabricated MTJ under DC = 10 μA exhibits two clear resistance states. The resistance of parallel (P) state is about 3.3 kΩ while that of antiparallel (AP) state is about 6.4 kΩ, i.e., tunnel magnetoresistance (TMR) effect. The lateral translation of magnetic field dependent resistance (R-H) curve is due to the stray field induced by the reference layer and synthetic antiferromagnetic layer. Hence, 500 Oe magnetic field is applied to counteract this stray field during the measurement of spin transfer torque (STT) induced magnetization switching to obtain a symmetric switching loop.

Fig. S1 (c) exhibits the current-voltage (I-V) curves of the MTJ under P and AP states. The I-V curve for P state is nearly linear while the I-V curve for AP state is significantly nonlinear, but there is no obvious rectification effect observed in either state. The I-V curve can be well described by a cubic equation[1],

$$I_{MTJ}(t) = A_1 V(t)^3 + A_2 V(t) \tag{1}$$

where A_1 and A_2 depend on the height and thickness of the tunnel barrier. By numerically fitting the I-V curves of MTJ, we can get A_1 and A_2 under P and AP states. As plotted in the inset of Fig. S1 (c), with increasing applied current, the TMR ratio decreases from 0.9 to 0.7 owing to the inelastic scattering induced by magnon excitation [2]. Here, the TMR ratio is defined as

$\mathrm{TMR} = \frac{V_{AP}-V_P}{V_P}$, where V_{AP} (V_P) is the voltage detected in AP (P) state. We also measure the I-V curve of Schottky diode as shown in Fig. S1 (d), where obvious rectification behavior with a forward opening voltage (V_{FO}) and a reversed breakdown voltage (V_{RB}) is observed. The inset plots the positive branch of I-V curve, which can be well described by the following exponential equation [3],

$$I_{Diode}(t) = I_s(e^{\alpha V(t)} - 1) \tag{2}$$

where I_s represents the reverse saturation current and $\alpha = \frac{nk_BT}{q}$. Here, n is the ideality factor and T represents temperature. By numerically fitting the I-V curve of diode, we can get α and I_s.

S2. Physical model and enhancement mechanism of on/off ratio

Fig. S2 (a) shows the basic structure of the combined device composed of 1 MTJ and 1 diode. The total current flowing through the combined device can be described as,

$$I(t) = I_{MTJ}(t) + I_{Diode}(t) \tag{3}$$

Meanwhile, the real time current applied to the combined device is

$$I(t) = I_1 \sin(2\pi f t) + I_2 \tag{4}$$

where I_1, I_2 and f represents AC amplitude, DC offset and AC frequency, respectively. Fig. S2 (b) illustrates the schematic of the AC with DC offset. The measured rectification voltage represents the average value ($\overline{V}$) of the corresponding real time voltage $V(t)$ over the period T of the applied AC current:

$$\overline{V} = \frac{1}{T}\int_0^T V(t)dt \tag{5}$$

Combining equations (3)-(5), the transport properties of the MLMU under AC and DC can be figured out, which are in good agreement with the experimental observations.

Moreover, the intermediate results under AC and DC can be calculated based on the physical model to clarify the enhancement mechanism of on/off ratio. Fig. S2 (c) illustrates the

instantaneous voltages and corresponding rectification voltages under AC = 20 μA and DC = ± 5 μA for P state. With DC offset changing from -5 μA to +5 μA, the rectification voltages are highly manipulated from negative to positive. Therefore, the rectification voltage under P state can be tuned to near zero through precisely regulating the proportion of AC and DC, high on/off ratio is realized. For example, Fig. S2 (d) illustrates the instantaneous voltage under AC = 20 μA and DC = 1.55 μA, which is tuned by the states of MTJ. For P state, the positive (green color) and negative (red color) integral areas are almost the same, leading to nearly zero rectification voltage of $\overline{V_P}$ = 0.0247 mV. For AP state, the instantaneous voltage for AP state within 5~10 presents larger enhancement than that within 0~5, resulting in a large rectification voltage $\overline{V_{AP}}$ = -8.43 mV. As a result, high on/off ratio, larger than 100, can be achieved. From the perspective of circuit analysis, this is because more current flowing through the diode is rectified to DC voltage under fixed AC when the MTJ changes from P to AP state.

S3. 3-State memory unit

Through changing the number of MTJs, different resistance states can be achieved. Fig. S3 (a) presents the schematic of the constructed 3-state memory unit through integrating 2-series MTJs and a Schottky diode in parallel. Fig. S3 (b) plots the magnetic field dependent rectification voltage under fixed AC amplitude of 3 μA with varying DC offsets. 3 stable resistance states and coordinate modulation are achieved. As summarized in Figs. S3 (c) and (d), higher than 100 on/off ratio is observed.

We also exhibit the working mode of the 3-state memory unit including data writing based on STT effect and data reading with high on/off ratio. As demonstrated in Fig. S4, multi-level rectification voltage under AC = 3 μA and DC = 0.14 μA corresponding the resistance states are stably detected when each MTJ is respectively switched from P state to AP state by locally applying a voltage pulse of 0.5 V to the corresponding electrodes.

S4. Crossbar array integration scheme of MLMU

For practical application, the proposed MLMU composed of series MTJs and a diode should be integrated in a crossbar array structure. Fig. S5 shows the designed crossbar array. The basic reading/writing operations are described as below.

On the one hand, through charging the selected bit line (BL) and word line (WL), the state of each bit cells (MTJs) can be changed, data writing operation is done. Taking the top-left MTJ as an example to describe the data writing operation. At first, the transistors P_0 and N_1 are turned on while the transistors P_1 and N_0 are turned off. Then, WL_0 and WL_1 are selected, the top-left MTJ is connected between BL_0 and BLB_0. After a pulse current, the resistance state of the selected MTJ can be changed by spin transfer torque (STT) effect. By this way, the resistance state of each MTJ in the crossbar array can be rewritten locally. Therefore, the weight for neuromorphic computing can be configured as the resistance states of the series-connected MTJs.

On the other hand, series-connected MTJs and a diode can be connected in parallel to realize high on/off ratio, and data reading operation is done. Taking the first column as an example to describe the data reading operation. The transistors P_0 and N_1 are turned on, transistors P_1 and N_0 are turned off while WL_0 and WL_{Diode} are selected. In this case, the 11 MTJs in this vertical line are connected in series, and then parallel connected with a diode. Therefore, the resistance of the MTJ can be read out with high on/off ratio through applying proper AC and DC component and detecting the rectification voltage. For a neuromorphic computing, the input signals can be encoded into different proportions of AC and DC components that exhibits high on/off ratio. Due to that the count of input signal is finite after the quantization, a look-up-table technique is used to store the one-by-one relation between input signal and AC/DC combination. Moreover, the output results of the array, i.e., rectified multivalued DC voltage, can be collected by using analog-to-digital conversion (ADC) circuit,

which has been verified by several works [4, 5]. In addition, an inverter can also be used to convert the voltage to the time domain, thereby completing the identification of the voltage signal with the advantages of high-speed and reliability [6].

S5. Integration density discussion

Considering that multiple MTJs are used to realize multi-level resistance states in our work, its integration density and improvement potential should be discussed, which is critical for practical application.

At first, the integration density of our proposed spintronic MLMU is comparable with that of magnetic random access memory (MRAM). To realize high-density integration, a crossbar array composed of large amounts of MLMUs is constructed as shown in Fig. S5. In this design scheme, a basic structure of 1 transistor and 1 MTJ is utilized. Therefore, the integration density of our scheme is compatible with that of MRAM. We can increase the numbers of resistance state through connecting more same MTJs in series and the state numbers equal 'n+1', 'n' is the number of MTJs. Furthermore, the integration density can be improved with the development of MRAM fabrication process.

Moreover, through precisely setting the junction resistance of each MTJ in the crossbar array, the equivalent states number provided by 1 MTJ (i.e., integration density) can be improved. The junction resistance of each MTJ can be configured to be different value through tuning the junction area, so the number of resistance states increases under same MTJ number. For example, 3 MTJs with same junction area provide 4 states in our experiment. If the junction area of each MTJ is different, 8 states can be realized. In brief, the number of states provided by 'n' MTJ increase from 'n+1' to '2^n', and the equivalent state number provided by 1 MTJ can reach '$2^n/n$'. With increasing 'n', the state numbers provided by 1 MTJ increases quickly because of the exponential growth. From this point of view, the integration intensity of proposed

MLMU with large ‘n’ can be comparable with that of 1-bit device with multiple states, such as memristor, i.e., basic cell of resistive random access memory (RRAM).

Reference

[1] W. F. Brinkman, R. C. Dynes, and J. M. Rowell, J. Appl. Phys. **1970**, 41, 1915.

[2] E. Y. Tsymbal, O. N. Mryasov, and P. R. LeClair, J. Phys.: Condens. Matter **2003**, 15, R109.

[3] D. Yang, F. Wang, Y. Ren, Y. Zuo, Y. Peng, S. Zhou, and D. Xue Adv. Funct. Mater. **2013**, 23, 2918.

[4] M. E. Sinangil, B. Erbagci, R. Naous, K. Akarvardar, D. Sun, W.-S. Khwa, H.-J. Liao, Y. Wang, and J. Chang, IEEE J. Solid-State Circuits **2021**, 56, 188.

[5] X. Si, R. Liu, S. Yu, R.-S. Liu, C.-C. Hsieh, K.-T. Tang, Q. Li, M.-F. Chang, J.-J. Chen, Y.-N. Tu, W.-H. Huang, J.-H. Wang, Y.-C. Chiu, W.-C. Wei, S.-Y. Wu, X. Sun, R. Liu, S. Yu, R.-S. Liu, C.-C. Hsieh, K.-T. Tang, Q. Li, and M.-F. Chang, IEEE J. Solid-State Circuits **2020**, 55, 189.

[6] Y. Zhang, J. Wang, C. Lian, Y. Bai, G. Wang, Z. Zhang, Z. Zheng, L. Chen, K. Zhang, G. Sirakoulis, and Y. Zhang, IEEE Trans. Circuits Syst. I **2021**, 68, 1193.

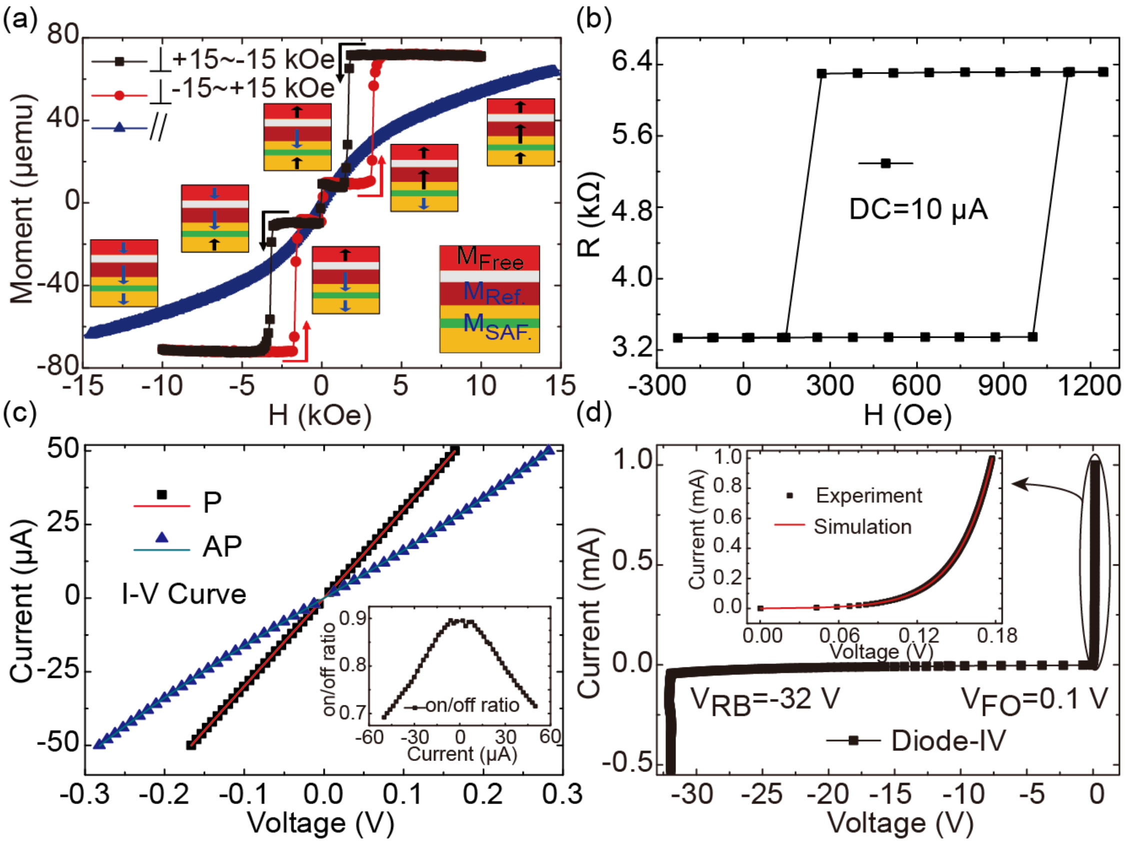

Figure S1. Basic properties of discrete components. (a) OOP and IP M-H loops of the multilayer membrane stack for fabricating MTJs. (b) R-H curve of the PMA MTJ measured under DC = 10 μA. (c) I-V curves of the PMA MTJ, which could be described by a cubic equation. Inset: the deduced on/off ratio under different current. (d) I-V curve of the Schottky diode. Inset: I-V curve under positive voltage, which can be well described by an exponential equation. In figure S1 (c) and inset of figure S1 (d), the scattering points are experimental data and the lines are theoretical simulation results.

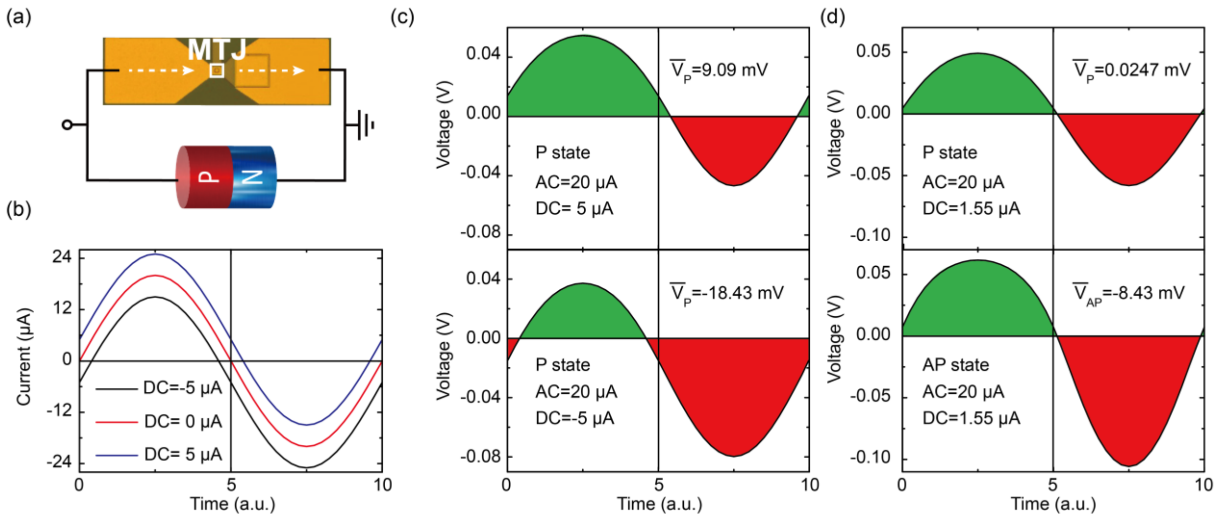

Figure S2. Mechanism of the on/off ratio enhancement. (a) Schematic of the parallel-connected MTJ and diode. (b) Schematic of the applied AC with DC offset. Simulated instantaneous voltages and corresponding rectification voltages (c) under AC = 20 μA and DC = ± 5 μA for P states and (d) under AC = 20 μA and DC = 1.55 μA for P and AP states. Green and red colors represent the positive and negative integral area, respectively.

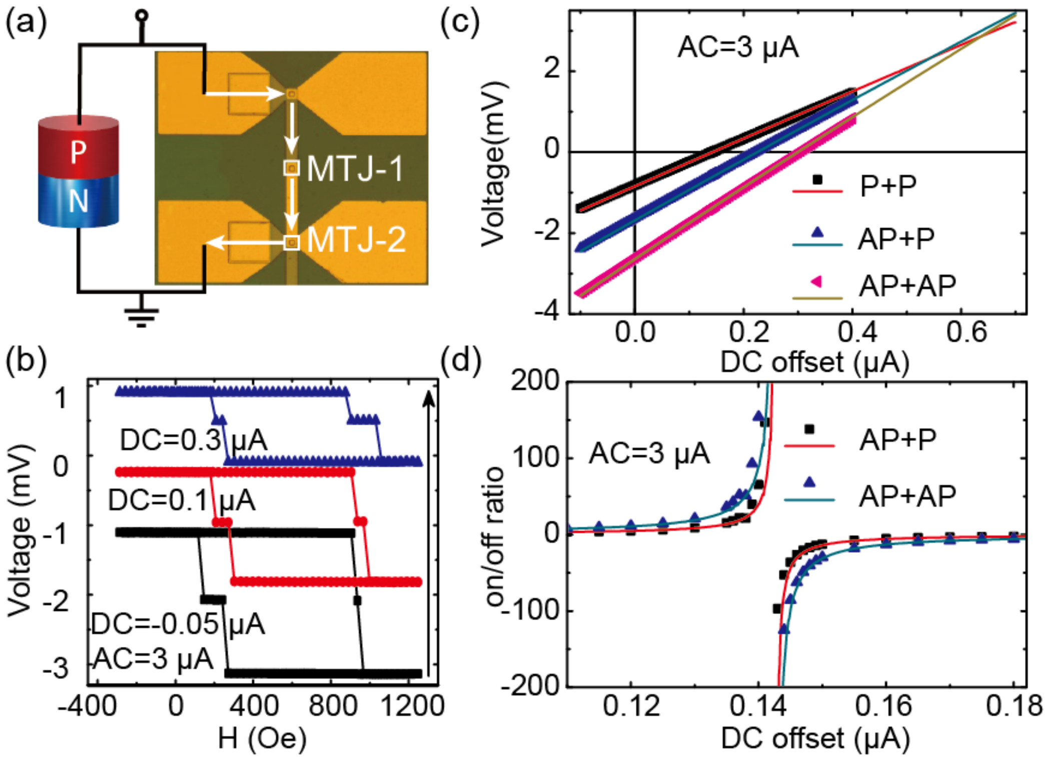

Figure S3. Transport properties of 3-state memory unit. (a) Schematic of 3-state memory unit. (b) Combined modulation of the multi-level rectification voltage by applying AC and DC. (c) Measured and calculated rectification voltage of each state modulated by AC and DC. (d) On/off ratio of the 3-state memory unit with AC and DC modulation. In figure S4 (c) and (d), the scattering points are experimental data and the lines are theoretical simulation results.

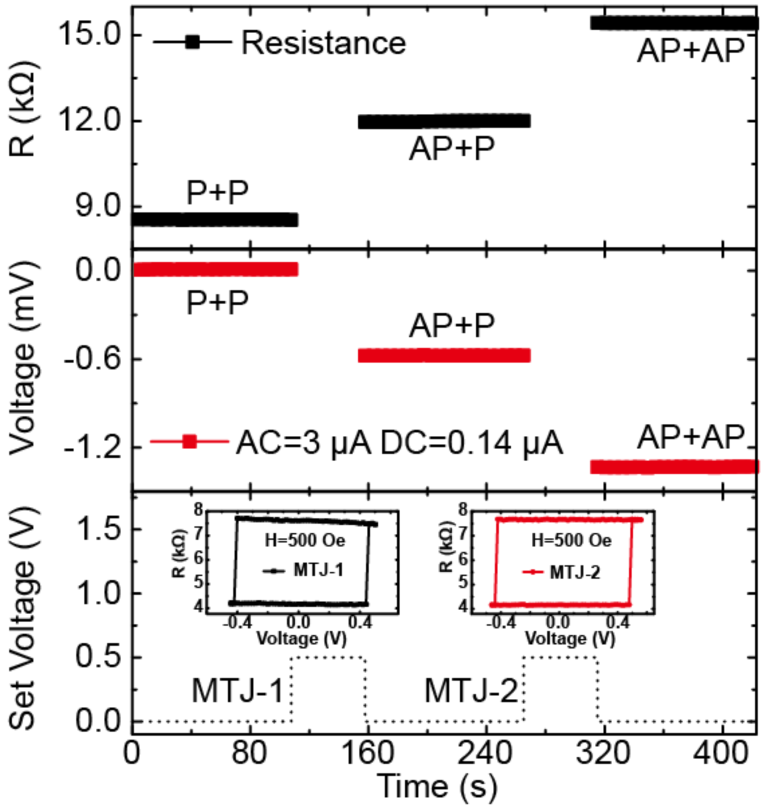

Figure S4. Operating process of the 3-state memory unit including data writing based on STT effect and data reading with high on/off ratio. Inset: STT-induced magnetization switching of each MTJ.

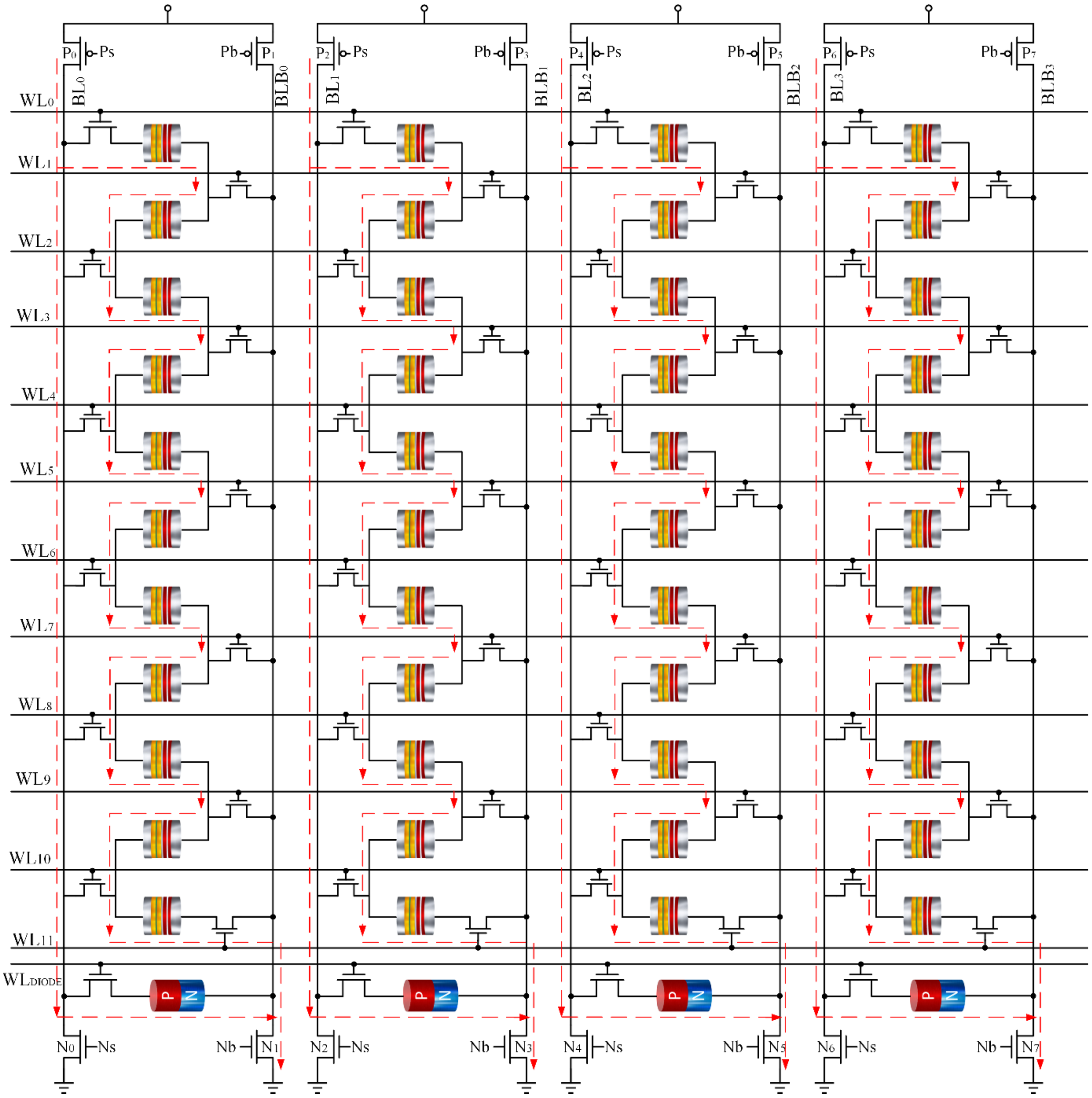

Figure S5. Crossbar integration scheme of our proposed MLMU.
